# Gastroenteritis Rehydration Of children with Severe Acute Malnutrition (GASTROSAM): A Phase II Randomised Controlled trial: Trial Protocol

**DOI:** 10.12688/wellcomeopenres.16885.1

**Published:** 2021-06-23

**Authors:** Peter Olupot-Olupot, Florence Aloroker, Ayub Mpoya, Hellen Mnjalla, George Passi, Margaret Nakuya, Kirsty Houston, Nchafatso Obonyo, Mainga Hamaluba, Jennifer A Evans, Roisin Connon, Elizabeth C George, Diana M Gibb, Kathryn Maitland

**Affiliations:** 1 Department of Paediatrics, Mbale Clinical Research Institute, Pallisa Road, Mbale, PO Box 291, Uganda; 2 Mbale Regional Referral Hospital, Pallisa Road, Mbale, PO Box 291, Uganda; 3 Department of Paediatrics, Soroti Regional Referral Hospital, Soroti, PO Box 289, Uganda; 4 Clinical Trials Facility, KEMRI-Wellcome Trust Research Programme, Kilifi, PO Box 230, Kenya; 5 Department of Medicine, Imperial College London, London, W2 1PG, UK; 6 Department of Paediatrics, University Hospital of Wales, Cardiff, Wales, CF14 4XW, UK; 7 MRC Clinical Trials Unit at University College London, University College London, London, WC1V 6LJ, UK

**Keywords:** Severe Malnutrition, Gastroenteritis, African Children, Intravenous fluids, WHO guidelines, Dehydration, Rehydration

## Abstract

**Background: **Children hospitalised with severe acute malnutrition (SAM) are frequently complicated (>50%) by diarrhoea ( *≥*3 watery stools/day) which is accompanied by poor outcomes. Rehydration guidelines for SAM are exceptionally conservative and controversial, based upon expert opinion. The guidelines only permit use of intravenous fluids for cases with advanced shock and exclusive use of low sodium intravenous and oral rehydration solutions (ORS) for fear of fluid and/or sodium overload. Children managed in accordance to these guidelines have a very high mortality. The proposed GASTROSAM trial is the first step in reappraising current recommendations. We hypothesize that liberal rehydration strategies for both intravenous and oral rehydration in SAM children with diarrhoea may reduce adverse outcomes.

**Methods **An open Phase II trial, with a partial factorial design, enrolling Ugandan and Kenyan children aged 6 months to 12 years with SAM hospitalised with gastroenteritis (>3 loose stools/day) and signs of moderate and severe dehydration.  In Stratum A (severe dehydration) children will be randomised (1:1:2) to WHO plan C (100mls/kg Ringers Lactate (RL) with intravenous rehydration given over 3-6 hours according to age including boluses for shock), slow rehydration (100 mls/kg RL over 8 hours (no boluses)) or  WHO SAM rehydration regime (ORS only (boluses for shock (standard of care)).  Stratum B incorporates all children with moderate dehydration and severe dehydration post-intravenous rehydration and compares (1:1 ratio) standard WHO ORS given for non-SAM (experimental) versus WHO SAM-recommended low-sodium ReSoMal. The primary outcome for intravenous rehydration is urine output (mls/kg/hour at 8 hours post-randomisation), and for oral rehydration a change in sodium levels at 24 hours post-randomisation. This trial will also generate feasibility, safety and preliminary data on survival to 28 days.

**Discussion**. If current rehydration strategies for non-malnourished children are safe in SAM this could prompt future evaluation in Phase III trials.

## Introduction

Annually, there is estimated to be 2.5 billion cases of acute gastroenteritis in children less than under five years of age. Gastroenteritis is reported to be the second largest cause of mortality in this age group and most deaths occur in low resource settings such as sub-Saharan Africa ^1^. A large case-control study Global Enteric Multicentre study (GEMS) conducted in Africa and Asia, examining the pathogenic aetiology and outcome of children admitted to hospital with moderate to severe gastroenteritis, found that they are 8.5 times more likely to die than children admitted without gastroenteritis (controls) ^2^. Thirty percent of the fatalities occurred less than seven days following hospitalisation and that baseline nutritional status an important factor in outcome. Improvements in early management may therefore be critical for improving outcome.

For children with severe acute malnutrition (SAM) the current World Health Organization (WHO) recommendations for rehydration of children with signs of severe dehydration and diarrhoea are exceptionally conservative and somewhat controversial ^3^. First, recommendations for intravenous rehydration are restricted only to those with advanced shock, a group we have documented to have an exceptionally high mortality rate (82% Day 28 mortality) on current guidelines since the guidelines focus on the exclusive use of oral rehydration using low-sodium oral rehydration solutions (ORS) (ReSoMal) ^4^. The guidelines lack relevant clinical studies, founded largely on a time-honoured and strongly held belief that the malnourished heart is at risk of heart failure. Moreover, guidelines indicate SAM children have an inability to cope with sodium-rich solutions (expert opinion, with no published primary data) and recommend a preference of hypotonic (half-strength Darrow’s or lactated Ringers with additional 5% dextrose) solutions for intravenous resuscitation ^5^. The controversy surrounding these recommendations was reviewed by Dr Brewster in 2006 (in a critical appraisal of the management of severe malnutrition) in which he states “The World Health Organization (WHO) manual has five pages on this issue (rehydration), which is somewhat unbalanced compared with the 11 lines on TB/HIV” ^3^. This is rather paradoxical since these were published there have been few studies to inform such recommendations, and the limited number of studies and trials suggesting that standard approaches are not harmful were not included in the WHO recommendations. Subsequent updates of these recommendations have also failed to include references to relevant research challenging these guidelines which we now review. 

### Challenging the WHO recommendations

First, these recommendations for rehydration and shock management are not physiological, since isotonic solutions are required to expand circulating volume, with hypotonic solutions more liable to cause water overload. Secondly, there are few commercially available preparations of half-strength Darrow’s or lactated Ringers with additional 5% dextrose (which the guidelines recognize) ^6^, but there is ready availability of half-strength Darrow’s and 50% dextrose. Reconstitution at the bedside is recommended but this risks contamination (infection) and medication errors (inaccurate glucose dosage calculation). Moreover, the recommendation that the reconstituted fluid should be used immediately is difficult to adhere in ordinary care. There is also a similar issue with the availability of low sodium rehydration solution (ReSoMal) which is frequently not stocked in resource limited hospitals as the sole producer of ReSoMal is Nutriset, France. Thus, as ReSoMal is infrequently available, even the oral rehydration aspect of these recommendations is impossible to implement. Finally, the views that ‘severe diarrhoea’ is unusual in SAM and the presence of diarrhoea is usual but of trivial consequence is not supported by scientific evidence ^7^. Furthrmore, guidelines indicating that signs of severe dehydration (decreased skin turgor or sunken eyes) are unreliable and reflect a lack of fat mass ^8^ lack a scientific published evidence-base and are at odds with published data ^7^. In two decades, guidelines have not been revised (including updated 2013 guidelines) ^5^ despite emerging evidence from Africa challenging these premises, where both diarrhoea and signs of severe dehydration were shown to have very high mortality rates ^7, 9^. These are summarised below.

### Data from Africa and systematic reviews

A prospective study involving 920 Kenyan children showed that 50% of children admitted with SAM had diarrhoea (case-fatality 21% versus 9% without diarrhoea) and a further 16% developed diarrhoea during admission (case-fatality 18%) ^7^. Key risk factors for death were bacteraemia and signs of severe dehydration (decreased skin turgor or sunken eyes) ^7^, and severe hyponatraemia - challenging the assumption that signs of severe dehydration are ‘unreliable’ and diarrhoea plays a limited role in the poor outcomes.

We have recently conducted two systematic reviews of randomized trials and observational studies in children with SAM and gastroenteritis with dehydration summarizing the evidence underpinning the intravenous and oral rehydration recommendations for SAM ^10, 11^

The intravenous rehydration review identified four studies which included 883 children, all of which were conducted in low resource settings ^10^. Two were randomised controlled trials ^12, 13^ and two observational cohort studies ^14, 15^. There was no evidence of fluid overload or other fluid-related adverse events including children managed on more liberal rehydration protocols. Mortality was high overall, particularly in children with shock managed on WHO recommendations (Day-28 mortality 82%). There was no difference in safety outcomes when different rates of intravenous rehydration were compared.

The Appropriate Fluid Resuscitation In Malnutrition (AFRIM) study incorporated systematic and detailed assessment of myocardial function demonstrating evidence of fluid responsive myocardial indices and that median systematic vascular resistance index remained very high post fluid resuscitation indicative of ‘under filling’ rather than overload ^15^. Another clinical study examining myocardial function in Kenyan children (CArdiac Physiology in MALnutrition [CAPMAL]) concluded that although cardiac mass is reduced (consistent with body mass) there were no significant differences in cardiac index, stroke volume index, and heart rate between inpatient children with and without SAM ^16^. This was also the conclusion of another larger study in Malawian children with severe malnutrition ^17^. 

For the management of ‘some dehydration’ the systematic review of oral rehydration ^11^ identified only two small clinical trials conducted in Asian children which have compared the use of ReSoMal against other low osmolar ORS ^18, 19^; both were equally effective in rehydration. However, both trials showed the development of hyponatraemia with ReSoMal, with one child developing seizures with severe hyponatraemia. Neither reported any mortality. No trials have been conducted in Africa, where SAM mortality remains high, with hyponatraemia as a key risk factor for mortality ^7^.

Finally, relevant to a broader population hospitalised with acute diarrhoea with severe dehydration ( ^~^10% weight loss) a prospective study showed that ~20% temporarily fulfilled anthropometric criteria for SAM (mid-upper arm circumference (MUAC) <11.5cm) but were ‘reclassified’ as undernourished ^20^ following rehydration. Thus, the current recommendations have much wider implications as potentially 20% of non-SAM children could be inappropriately rehydrated in accordance with the WHO SAM guideline. This may have contributed to the poor outcomes observed in the GEMS study ^2^.

## Protocol

Trial Registration ISRCTN76149273 (registered on 8 August 2018)

Protocol Version 2.1 Date 11 ^th^ August 2020

### Justification for the study

The principal aim of GASTROSAM is to reappraise these recommendations and evaluate more liberal strategies for both intravenous and oral rehydration (since they are interlinked as a ‘treatment bundle’). Demonstrating that intravenous rehydration is safe in children with SAM will be an important achievement.

Outcome remains poor in children hospitalised with diarrhoea, especially those with moderate and severe malnutrition. There is an urgent need to reappraise both the rehydration guidelines for both SAM and non-SAM patients owing to the very poor outcomes. Although numerous studies have indicated concerns over clinical assessment of degree of dehydration a number of census statements have considered these including European Society for Paediatric Gastroenterology Hepatology and Nutrition (ESPHGAN) ^21^ and National Institute for Health Care and Excellence (NICE) ^22^ guidelines indicating similar definitions for severe dehydration (with NICE indicating signs of shock to be included). Both review groups recognized that volume of 100 mls/kg for deficit replacement is probably correct for severe dehydration ( ^~^10% loss) but the evidence supporting rate of rehydration is very poorly informed with both rapid or ultra-rapid recommendations superseding a previous recommendation of rehydration over a period of 24 hours. NICE guidelines indicated an urgent need for “RCTs to provide cost effectiveness and safety of rapid versus slow rehydration” ^22^. 

The overarching aim of GASTROSAM is to generate the relevant evidence for refinement of future guidelines. Potential refinements anticipated include which children benefit from intravenous rehydration, the rate of rehydration and whether children with severe malnutrition should be managed by the same guideline as other children or by a dedicated guideline.

### Our hypotheses

For children with SAM with severe dehydration we hypothesize that the standard intravenous rehydration regime, known as WHO Plan C ^6^ (100mls/kg over three to five hours) recommended for non-SAM gastroenteritis with severe dehydration will result in better outcomes than the current very conservative SAM rehydration recommendations. In addition, we propose that the rate of rehydration may be critical and hypothesize that 100mls/kg over eight hours in SAM children could result in fewer fluid related adverse effects than the rapid WHO Plan C guidelines. We also propose that standard ORS solutions may be equally as effective with fewer side effects than lower sodium containing ORS for malnutrition (ReSoMal).

## Objectives

### General objective

To investigate:

(i) the current standard WHO rehydration protocol Plan C usually used in non-SAM children.

(ii) a slower rehydration regimen using the same total volume (100ml/kg of Ringers Lactate) over eight hours, irrespective of age

(iii) the current WHO restrictive intravenous rehydration strategy for SAM children.

In children with diarrhoea complicated by moderate or ‘some’ dehydration (see study population) and as follow on rehydration post-intravenous rehydration in those with severe dehydration whether oral rehydration with

(i) WHO standard oral rehydration solution (ORS) for non-SAM ^6^ is safer and results in less hyponatraemia and better outcomes compared to

(ii) current recommendation advocating low sodium ORS (ReSoMal) ^5^

### Specific objectives

(i) Document adverse events, particularly related to cardiovascular compromise and neurological sequelae.

(ii) To examine the feasibility (and adherence to) each of the strategies proposed

(iii) Gather a series of clinical and biochemical data on:

a. Initial assessment of dehydration
b. Response to treatment of children by intravenous rehydration.

(iv) To inform robust definitions of outcomes for a larger phase III trial

## Methods

### Study sites

The trial will be conducted at three centres; the Mbale Regional Referral Hospital, Uganda, the Soroti Regional Referral Hospital, Uganda, and the Kilifi County Hospital, Kilifi, Kenya.

### Study design

A Phase II trial to generate feasibility, safety, and preliminary data on survival to seven days and 28 days of rehydration strategies incorporating two strata for management of (A) severe dehydration comparing both rates of rehydration and volume of intravenous fluid replacement and (B) the composition of ORS for children with some dehydration and for children post intravenous rehydration. 

## Study populations

### Inclusion criteria

Children with SAM criteria (defined as these conditions: mid-upper arm circumference (MUAC) <11.5cm, WHZ <-3SD or kwashiorkor) ^6^, aged six months to 12 years, with gastroenteritis (> three loose stools/day):

### Severe dehydration: Stratum A

Signs of severe dehydration (as per WHO definition two or more of: unable to drink; AVPU <A (alert, voice, pain, unresponsive: system of recording patient’s level of consciousness); with sunken eyes; skin pinch goes back slowly (>2 seconds); and an inability to take or retain oral fluids), with or without shock. Shock will be defined by the recent 2016 WHO emergency triage assessment and treatment criteria; a patient with all of the following: cold peripheries with a weak and fast pulse (rate not specified) and a capillary refill time >3 seconds ^6^.

### Moderate/some dehydration: Stratum B

Defined as two of the following signs: restlessness or irritable; child is thirsty; child has sunken eyes; and skin pinch goes back less slowly(<=two seconds).

### Exclusion criteria

Diarrhoea lasting more than 14-days

Known congenital or rheumatic heart disease

Refusal of consent from parents/guardians of patient

## Sampling

### Sample size determination

In GASTROSAM, sample size calculations have been based on studies conducted in children with malnutrition and diarrhoea. Two African studies have reported on urine output (a surrogate for rehydration and perfusion status) in response to intravenous rehydration. Akech *et al.* reported persistence of oliguria (urine output <1ml/Kg/hour) at eight hours was more common in those receiving the WHO regimen with hypotonic solution (mean volume 30ml/Kg, with 9/22 (41%) as oliguric) versus children in isotonic Ringers Lactate arm (mean volume 39ml/Kg, 3/25 (12%) as oliguric), p=0.05 ^14^. In the second study using RL for rehydration oliguria was present in 3/11 (27%) on WHO regime vs 2/9 (22%) standard rehydration fluid-resuscitation ^15^. Two studies, both conducted in Asia, examined changes in sodium levels in response to oral rehydration with ReSoMal, with no fatalities in either of the trials ^18, 19^. In WHO ORS group, 1/64 (2%) developed severe hyponatraemia (sodium ≤120mmol/L) versus 3/62 (5%) in those receiving ReSoMal. Sodium levels were similar at baseline in both study arms but was lower at 24- and 48-hours in ReSoMal group than the WHO ORS group (p<0.01 and p<0.001 respectively) ^18^. A trial comparing ReSoMal versus hypo-osmolar ORS found a greater proportion of children receiving ReSoMal developed hyponatraemia (15.4% vs. 1.9%, p=0.03) ^19^.

Based on these data we calculated that for the Stratum A, a randomisation of 136 children would give 80% power (with 1:1:2 randomisation), with 34 to the rapid WHO Plan C arm, 34 to slow rehydration arm (100mls/kg over eight hours), and 68 to the WHO SAM arm to detect a difference of 2mls/kg/hr urine output at eight hours assuming a standard deviation (sd) of 4mls/kg/hr and mean of 2mls/kg/hr with one comparison between liberal fluid (Plan C and slow arm (100mls/kg over eight hrs) combined) and the WHO SAM arm. Including an additional 64 children with some dehydration in Stratum B (1:1) would give 80% power to detect a 3mmol/L difference in sodium levels (assuming mean of 132mmol/L and sd of seven) at 24 hours between the ReSoMal and ORS randomisation (total n=200, 100 per arm (32 some dehydration, 68 severe dehydration)).

### Study methods and procedures

All children admitted at the three centres with an acute history of gastroenteritis will be screened for study inclusion by the paediatric triage/admission team. All eligible children will be admitted to designated areas (so children can be closely monitored) in Mbale, Soroti, and Kilifi hospitals’ acute paediatric wards and if parents/guardian give consent to participate, children will be randomised to their treatment allocation by study clinicians (nurses or doctors) using cards in opaque sealed envelopes. The clinical procedures and laboratory investigations are summarised in Table 1 ^23^.

**Table 1.** Schedule of clinical assessments and laboratory investigations ^23^.

Hours (h) /Days (d)	0*h	8h	24h	7d	28d
Consent and information sheet	X				
Clinical examination (doctor/doctor visit)	X	X	X	X	X
Nurse observation/visit		X	X	X	X
Vital observations, anthropometry	X	X	X	X	X
Laboratory investigations					
Haematology	X		X	X	X
Biochemistry (Chemistry and Osmolarity)	X	X	X	X	
Lactate/Glucose	X	X	X		
Malaria slide + /- RDT	X		X	X	X
Blood culture	X				
HIV testing	X				
Urine (dipstick and save)	X	X	X	X	
Cross match (for transfusion) (red top) if indicated	X				
Stored samples					
Plasma for Cardiac Biomarkers	X	X	X	X	
Urine for osmolarity	X	X	X	X	

### Consent process

Once eligibility has been confirmed, authorized trial staff will approach parents/guardians to invite their child to take part in the trial. An information sheet will be provided to the parent/guardian in their usual language containing details of the GASTROSAM trial. The sheet will be read aloud to those who are unable to read. The doctor/nurse will check that the information has been fully understood and parents/guardians will be encouraged to ask questions they may have about their child’s participation in GASTROSAM. The information sheet and consent include details of the clinical trial, follow up, and additional biological samples taken for the trial and permissions for sample storage. Where possible, prospective written informed consent will be sought from parents/guardians who will then be asked to sign the consent form ^24^. Consent will include permission for the collection of admission and follow-up blood samples for later aetiological investigations. If parents/guardians are unable to sign, a thumbprint will be taken in lieu of a signature. A copy of the consent form will be given to the parent/guardian, the original placed in the patient’s medical notes, and a copy kept in the investigator site file.

A number of children will present as emergencies where delay in study enrolment, and thus treatment, will not be practical or indeed humane. We will use a modified form of deferred consent, developed for the FEAST (Fluid Expansion As a Supportive Therapy) trial ^25^ which we have now received ethical approvals for 26). This involves a ‘two-stage’ consent process where verbal assent is initially sought from parents or guardians by the admitting medical team, if it is considered that the full consent process would significantly delay treatment allocation, and consequently could be detrimental to the child’s health. Full consent is obtained once the child’s clinical condition has been stabilized. Caregivers will be provided with a brief verbal description of the trial and will be given the opportunity to ‘opt out’ of clinical. As in the FEAST trial, if following an assent process a child died prior to full written consent, full consent would not be sought.

### Treatment allocation

**Figure 1.**
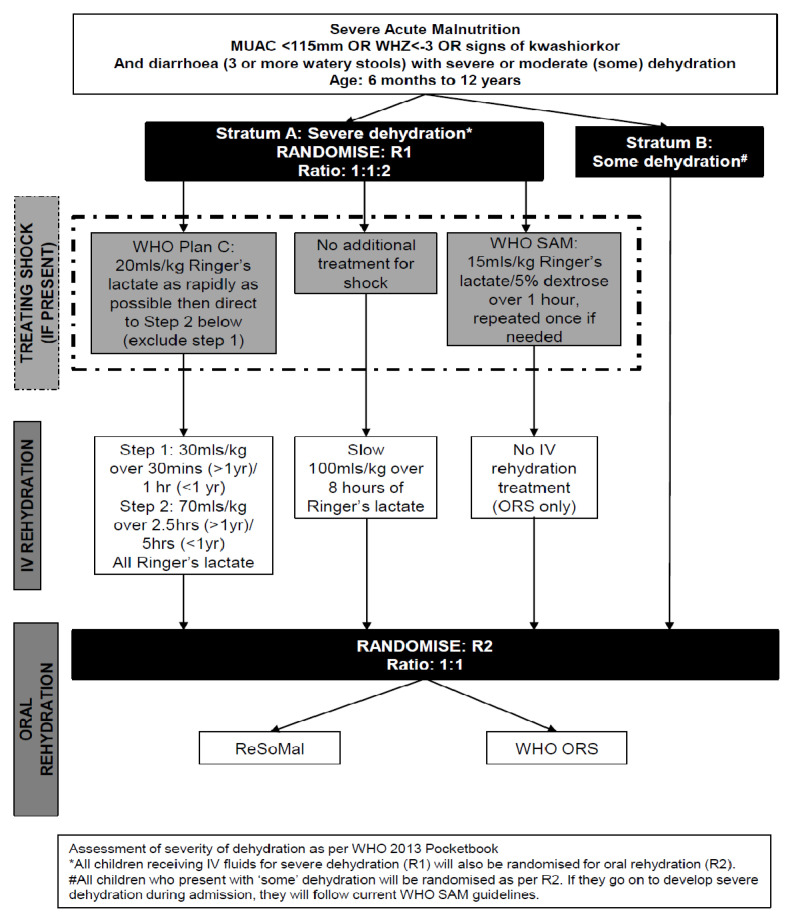
Trial flow.

Following consent, children will be randomly allocated to the treatment arms. Children in Stratum A with severe dehydration will be simultaneously assigned two randomisations (one for the intravenous rehydration strategy in a 1:1:2 ratio) and one for the oral rehydration strategy (1:1 ratio)). Children in Stratum B will be randomised to oral rehydration strategies (1:1 ratio). See Trial flow Figure 1. Randomisation lists will be generated stratified by site and stratum using permuted blocks of variable sizes and kept at the Medical Research Council (MRC) Clinical Trials Unit at University College London, London. Opaque sealed envelopes containing the randomised allocation will be prepared before the trial at the Clinical trials facility, KEMRI Wellcome Trust Research Programme (KWTRP), Kilifi. These will contain the actual allocation visible only once opened. The cards will be numbered consecutively within each stratum and opened in numerical order. Clinicians will be aware of the treatment-group assignments, but the laboratory tests are to be performed in a blinded manner.

### GASTROSAM Stratum A (Intravenous rehydration and ORS)

Signs of severe dehydration treatment Arms 1:1:2

i) WHO Plan C arm: Rapid intravenous rehydration as per WHO Plan C (usually for non-SAM children) (100mls/kg Ringers Lactate (RL) over three – six hours according to age including boluses (20mls/kg) for those with shock)
ii) Slow Rehydration Arm: A slower intravenous rehydration regimen (100 mls/kg RL given over eight hours and no boluses).
iii) WHO SAM arm: rehydration regime: ORS and intravenous boluses of RL only for shock (standard of care)

A second (simultaneous) randomisation for children in this stratum will be to one of two oral rehydration solutions received by children in Stratum B (see below).

Children presenting with some (moderate) dehydration (defined as two of restlessness or irritable; thirsty, sunken eyes or skin pinch goes back slow (<=two seconds) and thus not in Stratum A will be randomised to one of two oral rehydration solutions (GASTROSAM Stratum B) (ratio 1:1 for both groups). 

### GASTROSAM Stratum B (ORS only)

i) Standard WHO ORS given for non-SAM (experimental) versus
ii) WHO SAM-recommended low-sodium RESOMAL

Both oral rehydration solutions will be given in accordance with WHO Plan B oral rehydration regime over four hours. Treatment will be given according to requirements during hospitalisation (and may include continuing or restarting ORS to replace on-going losses or intravenous rehydration if there’s development of severe dehydration). All children can start ORS as soon as they are able to take and retain oral fluids, even before intravenous rehydration is complete (which is replacing deficit). For children unable to take fluids orally we will consider temporary placement of nasogastric tubes. 

### Clinical management and monitoring

Following correction of severe dehydration (based on a review of WHO clinical signs and observations), children will be assessed for their ability to take oral rehydration or feeds. Children who are able to take and retain oral fluids/feeds and who are in neutral or marginally positive fluid balance (both input and output will be measured) will be considered as satisfactorily rehydrated.

All children will be offered oral rehydration fluids alongside their intravenous rehydration regimen; in GASTROSAM this will follow the randomised treatment allocation. Each child will have an input-output monitoring chart, i.e., including urinary catheter volumes and diaper weights, to document the volumes that children in both arms are drinking and retaining as well as defining clinical endpoints that will be used to guide when to stop intravenous fluids in future studies. For the purposes of this study, each child will aim to complete their allocated intravenous fluid hydration regimen.

### Management of potential complications

Children enrolled in GASTROSAM will be very closely monitored, using the same systems for adverse event reporting used in the fluid resuscitation, FEAST trial ^25^, and the transfusion TRACT trial ^27^ that examined more liberal volumes of fluid and blood respectively than was currently recommended enrolling at the centres involved in the current GASTROSAM trial. Prior to the start, the study teams will undergo detailed training and clear guidelines on what signs to look for and how to treat suspected fluid overload or heart failure will be included in the manual of operations, which are similar that which were developed and implemented in the FEAST trial ^25^. Irrespective of which arm of the trial patients are included in, the treatment of life-threatening side effects would be assured. This would include prospective monitoring for pulmonary oedema or signs of cardiac failure or the development of severe hypotension which would be treated with a fluid bolus therapy to restore systolic blood pressure. Mechanical ventilation is not available any of the hospitals which largely reflects the typical situation in most hospitals in sub-Saharan Africa. In the case of a child who develops clinical signs and fluid overload, the management plan is to stop intravenous fluids and, if there are clear signs of pulmonary oedema/heart failure, to administer intravenous frusemide and oxygen, monitoring the child closely with hourly observations until stable and further fluid management to be administered orally (or via nasogastric tube if the child is unable to take fluids orally). 

If, after the initial rehydration regimen is completed, there are ongoing significant gastrointestinal fluid losses, this will be managed according to clinical signs which, if severe dehydration or shock develops may include one repeat of intravenous regime previously allocated protocol and then, personalised fluid management to take account of input/output.

## Clinical endpoints

### Primary outcome

For intravenous rehydration: urine output (mls/kg/hour at eight hours) as a surrogate maker of rehydration efficacy.

For oral rehydration strategy: change from randomisation in sodium levels at 24 hours. 

### Secondary outcomes

Measures assessing safety (evidence of pulmonary oedema or heart failure); change in plasma sodium levels from baseline (time of enrolment) to completion of intravenous rehydration levels for those in Stratum A; perturbations of electrolyte abnormalities (severe hyponatraemia <125 mmols/L or hypokalaemia <2.5 mol/L), weight and MUAC change to Day seven; and Day-28 survival.

All children will be catheterized in order to achieve accurate fluid balance calculations. The urinary catheter can be removed after eight hours (primary endpoint). All children will have a daily weight and fluid balance calculated until discharge (performed at the same time each day) and at follow up. All children will be followed up on Day seven (post discharge). They will be reviewed by a clinician, have their weight and observations recorded. All children will also have one further blood sample (biochemistry and cardiac enzymes) taken on Day seven. Follow up clinical assessments of hydration (in children without any on-going losses or intercurrent illness) serve to validate inpatient assessments of hydrations status. Prior to the day of follow up the parents will be contacted by the study site coordinators to remind them of the clinic visit. This system has previously been used in the FEAST and TRACT trial and lead to very high study retention and follow-up ^25, 27^.

Clinical and haemodynamic (vital signs) responses will be monitored every 30 minutes for the first two hours then hourly until eight hours, and every four hours up to 24 hours and then every 12 hours until discharge, as will pre-specified serious adverse events (SAEs) of interest:

a) new onset seizures or worsening conscious level

b) signs of pulmonary oedema

c) and/or cardiac failure after the initiation of intravenous rehydration

Time to tolerate oral fluids/feeds and maintenance of normal fluid balance and time to discharge will also be collected

### Adverse events and interim analyses

SAEs will be reviewed immediately by a designated physician (SAE reviewer) and reported to the appropriate ethics and regulatory committees within one week. Severe adverse events will use the standardized definitions and external review process of these events will be carried out using the same criteria as were used in the FEAST trial. The Chief investigator will inform the Trial Steering Committee (TSC) and Data Monitoring Committee (DMC) for review on a regular basis (as deemed necessary).

### Data Monitoring Committee

An independent Data Monitoring Committee (DMC: see composition at the end of the protocol) will review data on enrolment, safety, adherence to the trial protocol, and efficacy at regular intervals and in strict confidence. The terms are covered in the DMC charter, signed by the chair (TP) and trial statistician (ECG). There are no fixed ‘stopping rules’. However, the DMC will receive and review information on the progress and accruing data of the trial and provide advice on the conduct of the trial to the TSC. The DMC would inform the Chair of the TSC if, in their view, the results are likely to convince a broad range of clinicians, including those supporting the trial and the general clinical community, that, on balance, one trial arm is clearly indicated or contraindicated for all participants or a particular category of participants. The DMC is comprised of a chair and three other independent members. None declared conflicts of interest.

### Auditing

The study will be monitored by the Trial Management Team from the Kilifi Clinical Trial Facility (KEMRI-Wellcome Trust) with the help of the Mbale Clinical Trial monitoring team. The monitors have been trained on the study protocol and will be familiar with all study procedures. During the initial visits, the monitors will review 100% of the fields of all the study forms. Subsequently the monitors will review 100% data contributing to the primary endpoint and 100% of fields for a randomly selected sample of study forms. All forms monitored during a visit will be detailed in the monitoring visit report. A database check for accuracy of data entry is performed at regular intervals. The data points are to be checked will be endpoint data and safety data. After site initiation visit (SIV) the first routine visit will be done after the first five participants are enrolled then the other routine visits depending on the rate of recruitment and issues raised during the initial visit the frequency of the routine visits will be decided thereafter. The SIV was done on 20 ^th^ August 2019 for the Mbale site and 28 ^th^ August for the Soroti site. The 1 ^st^ routine monitoring on site visits were done on 4 ^th^ and 5 ^th^ Feb 2020 respectively. At the time of the writing the protocol manuscript, the Kilifi site had not commenced enrolment as it was pending regulatory approval. 

### Data management

All clinical and laboratory data will be recorded in the case report form and stored with a unique serial number identifier. Data will be entered (double data entry) onto the Open Clinical database. All data will be regularly backed up and backup copies stored both on and off site. Paper records will be archived in locked cabinets at the respective study sites. These cabinets will have limited access with prior authorisation (by the site principal investigators). All data will be partially- anonymized prior to presentation or publication of any results. Study participants will be identified by a unique subject identification number, but patient identifiable information will not be recorded on the study database in compliance with good clinical practice (GCP) requirements. The data will be examined for inconsistencies during the trial by the statistician and fed back to study sites for corrections following GCP procedures.

### Confidentiality

All clinical data will be held confidentially, and personal identifiers will be removed before analysis of the data and presentation of the results.

### Data sharing

After completion of the study, requests for data access from researchers outside the study team will be considered by the trial management team and clinical trials unit (Data Governance Committee), and where indicated, requestors will be asked to develop scientific protocols for approval of secondary analyses. The potential to share data will be included in the participant Information and Consent Form ^24^.

### Statistical analysis

The primary analysis for the trial will describe baseline parameters (stratified by study stratum and arm), time to tolerate oral fluids/feeds and maintenance of normal fluid balance and time to discharge, and compare primary and secondary endpoints by trial arm. The primary analysis will be by intention to treat. The primary endpoint of urine output (mls/kg/hr by eight hours) will be compared between arms with a t-test assuming unequal variances between the arms, and will compare the WHO SAM arm to the other two arms (WHO Plan C arm and Slow rehydration arm) combined. 

A secondary analysis will use pair-wise comparisons of urine output using a t-test between each arm and the standard of care (WHO SAM arm). The primary endpoint for the oral rehydration comparison of change in sodium at 24 hours will be analysed using normal linear regression, adjusting for baseline (measured at randomisation), with appropriate transformations if necessary.

The secondary endpoint of change in sodium at 24 hours from post-intravenous levels for those in stratum A will also use normal linear regression, adjusting for the value measured after the end of their intravenous rehydration. Changes in weight, MUAC and electrolytes will be analysed using normal linear regression (potentially on log-transformed data), and generalised estimating equations to jointly model changes during admission and at day-seven. Analysis of adverse events, including mortality, evidence of pulmonary oedema and heart failure, will use time to event methods through day 28 counting in hospital death as a competing risk. Adverse events will also be summarised by body system. Analysis of perturbations of electrolyte abnormalities (severe hyponatraemia (<125mmols/L) or hypokalaemia (<2.5mol/L) will use time to event methods through day 7 counting in hospital death as a competing risk. 

## Ethics statement

Ethical approval has been obtained Mbale Regional Hospital Research Ethics committee (MRRH_REC OUT 107/2018) for both Mbale and Soroti Regional Referral Hospital sites, from KEMRI Scientific and Ethics Review Unit (SERU) for the Kilifi County Hospital site (KEMRI/RES/7/3/1) and from Imperial College Research Ethics Committee (18IC4427), the sponsor of the study. The trial has been registered on ISRCTN (registry identifier 76149273) on 8 ^th^ August 2018 (Version 1.0) and the Pan African Clinical Trial Registry ( www.pactr.org) database (identifier PACTR202103852542919) on 23 ^rd^ March 2021 (Version 2.0). 

### Safety

The study will be performed in patients who may potentially benefit from the treatment. The risks of cannula insertion and blood drawing include pain, infection at the site of the cannula, and thrombophlebitis. These will be minimised by careful technique according to a standard SOP, cannula site inspection, and replacement or removal where necessary. No more than 1ml/kg of blood will be drawn for research at any one time ^10^.

Whilst there is a concern that liberal fluid rehydration may cause heart failure, our recent publication on myocardial function in this population given rehydration did not show this resulted in heart failure ^16^. Children enrolled in GASTROSAM will be very closely monitored, using the same systems for adverse event reporting used in the FEAST trial ^25^ and the transfusion (TRACT) trial ^28^ both examined more liberal volumes of fluid and blood respectively than was currently recommended. Blinded SAEs (removing all references to intervention arm) will be independently adjudicated by an endpoint review committee.

In neither the FEAST nor the TRACT trial were any concerns raised by the data monitoring committee that more liberal volumes were causing fluid overload. GASTROSAM will be conducted in three of the trial sites for FEAST and TRACT and are familiar with the conduct of trials in critically sick children. All study teams will undergo detailed training and included in the manual of operations will be clear guidelines on what signs to look for and how to treat suspected fluid overload or heart failure, which were developed and implemented in the FEAST trial.

### Benefits

All patients will be closely monitored so that clinical deteriorations can be identified at the earliest opportunity and appropriate therapy initiated. In general, the trial sites in Africa have considerable experience with this population and this will serve to minimise the risks to the patients and the trial ^10^. Prior to the start, the dedicated study teams will undergo detailed training on general management of SAM and its complications and receive very detailed training on fluid management. This will be included in the manual of operations which will provide clear guidelines on what signs to look for and how to treat suspected fluid overload or heart failure, which were developed and implemented in the FEAST trial. We believe this will afford all children enrolled in the trial with a higher quality of care. 

## Plans for dissemination of the study outcomes

### Public engagement

Results from this trial will be disseminated locally through community meetings and national meetings with the wider healthcare professional community. These systems have been developed for dissemination of MRC FEAST and TRACT trial results and will be extended to include GASTROSAM.

### National policymakers

The site principle investigators will discuss with their Ministry of Health about the ongoing trial. A summary or briefs will be produced to highlight the trial results and next steps required to inform rationale evidence-based guidelines.

### International policymakers

We have already had teleconferences with members of the epidemic consortium International Severe Acute Respiratory and Emerging Infection Consortium (ISARIC) and WHO GOARN (Global Outbreak Alert and Response Network), UK Rapid Response Teams, and MSF International Coordinator to share the systematic reviews we undertook in preparation of the study outline proposed ^11, 29^. The current rehydration management guidelines for children with severe malnutrition are under intense speculation currently as a potential reason for the poor outcome in the current cholera epidemics. We have already shared the results of the GASTRO phase II trial ^30^ with these bodies. Through these connections, we will seek meetings with WHO, the United Nations Children’s Emergency Fund (UNICEF), and other international policy makers to discuss the results and subsequent trial plans.

## Discussion

The principal questions being addressed in the GASTROSAM trial are driven by the equipoise over fluid management of children with SAM. It is also informed by the very poor outcomes of African children hospitalised with SAM, that is commonly complicated by diarrhoea ( ^~^50%), where case fatality (CF) is 21%, and even worse if also complicated by severe dehydration (CF 39%). Yet, WHO management guidelines fail to recognise diarrhoea as anything other than of ‘limited consequence’ with strong warnings to practitioners that signs of dehydration are spurious (based on expert opinion). The guidelines also consider that children with SAM are considered at high risk of cardiac failure and sodium overload, but until recently the evidence to either support or challenge these views has been limited. In the last few years, these views have been challenged by two seminal publications reporting on cardiac function in African children hospitalised with SAM ^16, 17^. One prospective case-control study in 88 Kenyan children with SAM (cases) and 22 non-malnourished hospital controls (matched by age and presenting clinical syndrome) demonstrated no evidence that children with SAM had features of cardiac failure, nor were children at greater risk of cardiac dysfunction or arrhythmias than controls. Moreover, the cardiovascular profiles of children with marasmus were similar to those with kwashiorkor ^16^. In contrast with the current guideline recommendations ^5^, children receiving intravenous rehydration or fluid boluses for shock show both clinical and echocardiographic findings indicating largely fluid-responsive changes without evidence of fluid overload. These finding are also supported by a large study of 272 Malawian children which demonstrated no significant differences in cardiac index, stroke volume index and heart rate between inpatient children with and without SAM ^17^. In summary, the two studies concluded that there is no overwhelming evidence in African children with SAM that biventricular failure is a concern (including those receiving intravenous fluid replacement) and thus justify the ethical conduct of trials examining more liberal rehydration strategies.

Our group recently published a trial (GASTRO) ^31^ comparing slow versus rapid Plan C (standard of care) rehydration (as proposed for this trial) in Kenyan and Uganda children with severe dehydration following gastroenteritis. Children with severe malnutrition were excluded from this trial. We enrolled 122 children and had only four deaths (two deaths in each arm (3% mortality overall) ^30^. Whilst the trial was not powered to examine superiority of one arm over the other, it did demonstrate that the experimental arm (slow rehydration) was safe and, when compared to historic controls, an unselected paediatric admission cohort with gastroenteritis and severe dehydration (n=1211) across a network of Kenya hospitals where gastroenteritis was managed on plan C management, in-hospital mortality was 12% ^32^.

As with the GASTRO trial ^30^, the main aim in GASTROSAM trial is to generate pilot safety data and surrogate markers of efficacy including urine output, correction of hyponatraemia, haemodynamic correction, and weight gain and some indicative survival data to inform the design and sample size calculations for a large overarching trial of rehydration for gastroenteritis/diarrhoea. If liberal intravenous strategies in GASTROSAM (and slower rates of rehydration in GASTRO) were shown to be safe we propose a future pragmatic Phase III trial enrolling children irrespective of nutritional status. For those with severe dehydration a 2-arm trial in non-SAM comparing rates of intravenous rehydration and for SAM a 3-arm trial comparing two rates to current standard of care. Included in a future trial would be a comparison of ORS to RESOMAL for SAM children with some dehydration and post intravenous rehydration. The primary outcome would be D7 and D60 mortality (aligned to GEMS endpoint) ^2^. Secondary outcomes include mortality, length of hospital stay, subsequent hospital readmission and adverse events.

## Trial status

Trial enrolment started on 2 ^nd^ September 2019. 

## Protocol version changes

Version 1.0 was the original protocol submitted for ethical approval to Imperial College Research Ethics Committee in February 2018 and approved (20 ^th^ March) following additional background information included in the Protocol (version 1.2: 5 ^th^ June 2019). Further clarifications were requested following review by MRRH_REC, Uganda Version 1.2 was approved by MRRH-REC on June 14 ^th^ 2019. Version 2.0 (23 ^rd^ Sep 2019) included 2 investigators, Dr. Damalie Nalwanga to be a co-investigator based in Mulago National Referral Hospital, Dr. Martin Chebet to be a co-investigator based in Mbale Regional Referral Hospital and Replaced Dr. Margaret Nakuya as co-investigator with Dr. Betty Adong for the Soroti Site. Version 2.1 dated 11 ^th^ August 2020_was prepared to include Kilifi County Hospital, Kilifi, Kenya as a replacement site for Mulago National Referral Hospital, which was removed from the protocol as they were unable to host the trial.

## Roles and responsibilities

### Role of study sponsor and funders

The sponsor and funder played no role in in study design and will play no role in data collection, trial management, analysis and interpretation of data and manuscript preparation the decision to submit the report for publication.

**Trial Management Group**

Kathryn Maitland, Peter Olupot-Olupot, Mainga Hamalubu, Hellen Mnjalla, Elizabeth C George and Roisin Connon

**Trial Steering Committee**

**Professor Elizabeth Molyneux**, OBE (Chairman): College of Medicine, Blantyre, Malawi 

**Dr Jane Crawley** University of Oxford, United Kingdom

**Dr Irene Lubega** Department of Paediatrics Makerere University College of Health Sciences, Uganda 

**Professor William Macharia** Department of Paediatrics and Child Health, Aga Khan Hospital, Nairobi, Kenya.

**Data Monitoring Committee**

**Prof. Timothy Peto** (Chair) University of Oxford

**Dr. Carolyne Ndila** (statistician) formerly based at KWTRP

**Professor Dr. Edison Mworozi** Department of Paediatrics, Mulago Hospital, Kampala, Uganda

**Professor Philippa Musoke** Makerere University College of Health Sciences, Kampala, Uganda.

**Endpoint Review Committee**

Jennifer Evans and Diana Gibb

## Data availability

### Underlying data

No underlying data are associated with this article.

### Extended data

Imperial College Research Data Repository. GASTROSAM Phase II trial: Patient information Sheet and Consent form. 10.14469/hpc/8308 ^24^.

This project contains the following extended data:

- Patient information Sheet and consent forms.

Imperial College Research Data Repository. Gastroenteritis Rehydration Of children with Severe Acute Malnutrition (GASTROSAM): A Phase II Randomised Controlled trial Trial Protocol. 10.14469/hpc/8143 ^23^.

This project contains the following extended data:

- Figure 1-Trial Assessment schedule. (Schedule of clinical assessments and laboratory investigations outlined in Table 1).

Data are available under the terms of the Creative Commons Zero "No rights reserved" data waiver (CC0 1.0 Public domain dedication).

### Reporting guidelines

Imperial College Research Data Repository: SPIRIT checklist for ‘Gastroenteritis Rehydration Of children with Severe Acute Malnutrition (GASTROSAM): A Phase II Randomised Controlled trial Trial Protocol’. https://doi.org/10.14469/hpc/8143 ^23^.

Data are available under the terms of the Creative Commons Zero "No rights reserved" data waiver (CC0 1.0 Public domain dedication).

## Abbreviations

FEAST      Fluid Expansion As a Supportive Therapy

GEMS      Global Enteric Multicentre study

KWTRP   KEMRI Wellcome Trust Research Programme

MRC        Medical Research Council

MUAC     Mid-upper arm circumference

ORS         Oral Rehydration Solution

ReSoMal  Low sodium Rehydration Salt Solution for Malnutrition

SAM        Severe acute malnutrition

WHO       World Health Organization
