# Supplementary material for: Gastroenteritis Rehydration Of children with Severe Acute Malnutrition (GASTROSAM): A Phase II Randomised Controlled trial: Trial Protocol
Source: Wellcome Open Res. 2024 Jan 16;6:160. Originally published 2021 Jun 23. [Version 2] doi: 10.12688/wellcomeopenres.16885.2 (PMC8276193; doi:10.12688/wellcomeopenres.16885.2)
Supplement: Peer review history

# Reviewer report: Reviewer response for version 1

**DOI:** 10.21956/wellcomeopenres.18626.r44660

**Published:** 2021-07-08

**Authors:** Roberta Petrucci

**Affiliations:** 1 Médecins Sans Frontières, Geneva, Switzerland

Management of dehydration in SAM children represents one of the top challenges in clinical practice in LIC. Despite being a highly preventable and treatable condition, we face a disheartening lack of evidence to properly manage dehydration, especially in SAM children.

 Currently available recommendations are associated with poor short- and long-term outcomes and, not surprisingly, in clinical practice divergences from standard treatment are common, but in the lack of evidence-based alternatives, practitioners are left without guidance and the risk of providing suboptimal care is not neglectable.

 Notably, in under resourced and overcrowded paediatric units in sub-Saharan Africa, simplicity, safety and feasibility of an intervention must be carefully evaluated along with its effectiveness. Current recommendations lead to complex decisional tree that stratifies patients according to age, nutritional status, level of dehydration, leading to many different therapeutic plans, increasing the possibility of misclassification and therefore exposing children to potential mismanagement.

 The GASTRO-SAM study is therefore extremely welcomed. With this trial, Prof Olupot-Olupot and his colleagues aim to provide structured and solid evidence that will inform clinical guidance.

 The study design is solid, transparent and easily reproducible with the information and resources provided. Safety endpoints, confidentiality, ethical issues are addressed by the clearly stated procedures and methodology and a monitor committee with external experts.

 Findings will be applicable widely as study conditions are similar to many hospitals in sub-Saharan Africa. If the hypotheses of the study are proven true and confirmed in phase III trial, solid evidence will be available to inform clinical guidance that will moreover benefit from simplification. One single parenteral rehydration regimen, regardless of age and nutritional status, will enhance uptake and implementation, while requiring less training and supervision. Reduction in the risk of misclassification, medical errors, mismanagement will improve safety. The possibility to use one single product for oral rehydration is another non neglectable advantage in terms of supply and end-user drug management.

Is the study design appropriate for the research question?

Yes

Is the rationale for, and objectives of, the study clearly described?

Yes

Are sufficient details of the methods provided to allow replication by others?

Yes

Are the datasets clearly presented in a useable and accessible format?

Yes

Reviewer Expertise:

Paediatric humanitarian medicine

I confirm that I have read this submission and believe that I have an appropriate level of expertise to confirm that it is of an acceptable scientific standard.

# Response

**Published:** 2021-07-09

**Authors:** Kathryn Maitland

**Affiliations:** KEMRI-Wellcome Trust Research Programme, Kenya

Thank you for the very positive review outlining the realities of implementing the current recommendations. We hope this trial will provide the relevant safety and feasibility data for a future Phase III trial.

# Reviewer report: Reviewer response for version 1

**DOI:** 10.21956/wellcomeopenres.18626.r44655

**Published:** 2021-06-29

**Authors:** C. Louise Thwaites

**Affiliations:** 1 Oxford University Clinical Research Unit, Ho Chi Minh City, Vietnam

- This is a study protocol for a phase II clinical trial comparing rehydration regimes for children with severe malnutrition and diarrhoea. This is an important clinical question where high quality evidence is lacking.
- The protocol follows SPIRIT guidelines and has been carefully thought through and builds on the extensive clinical trial experience of this group in similar context. Importantly this includes very specific methods for detecting and evaluating safety endpoints.
- The trial has received the appropriate ethics and regulatory approvals and is currently ongoing. This is a well-considered protocol addressing an important clinical question and I have no comments.
- A minor point for the authors is whether the trial status section in the discussion should be expanded to clarify whether recruitment is currently ongoing or suspended.

Is the study design appropriate for the research question?

Yes

Is the rationale for, and objectives of, the study clearly described?

Yes

Are sufficient details of the methods provided to allow replication by others?

Yes

Are the datasets clearly presented in a useable and accessible format?

Not applicable

Reviewer Expertise:

Clinical trials; resource-limited critical care

I confirm that I have read this submission and believe that I have an appropriate level of expertise to confirm that it is of an acceptable scientific standard.
